# When the Minority Thinks “Essentially” Like the Majority: Blacks Distinguish *Bio-Somatic* from *Bio-Behavioral* Essentialism in Their Conceptions of Whites, and Only the Latter Predicts Prejudice

**DOI:** 10.1371/journal.pone.0160086

**Published:** 2016-08-04

**Authors:** Michael J. Gill, Dana M. Mendes

**Affiliations:** Department of Psychology, Lehigh University, Bethlehem, Pennsylvania, United States of America; University of Melbourne, AUSTRALIA

## Abstract

Essentialist beliefs about social groups can contribute to prejudice and intergroup distancing. To date, little data have been gathered regarding minority group members’ essentialistic thinking about the White majority in the U.S. Do essentialist beliefs show a similar structure when minority group members are thinking about the majority as when the majority group is thinking about the minority group? Do minority group essentialist beliefs predict affective prejudice and diminished desire for intergroup contact as they do among White respondents? We sought answers to these questions in a study that included 248 African American participants. We found clear evidence that the structure of Blacks’ essentialist thinking about Whites matches the structure of Whites’ essentialist thinking about Blacks. Specifically, Black respondents made a distinction between bio-somatic and bio-behavioral essentialism, and reported stronger endorsement of the former as compared to the latter. Also replicating prior studies of Whites’ essentialist thinking, only bio-behavioral essentialist beliefs were predictive of negative attitudes. This suggests that essentialism can be linked to prejudice even in contexts that do not involve a dominant group rationalizing its social advantages. Discussion centers on implications of this work for prejudice reduction.

## Introduction

Because of its likely role in intergroup prejudice and violence, social psychologists have sought to understand the role of essentialist thinking in lay conceptions of social groups. Many scholars have focused on the question of what, precisely, it means to hold essentialist beliefs about a social group [1–5]. Others have focused on possible attitudinal consequences of holding essentialist beliefs, including affective prejudice [5–8] and attitudes toward contact with the outgroup [9].

Overwhelmingly, the empirical data explore the essentialist thinking of the dominant Caucasian majority in Europe and the United States. Although a handful of scholars have studied Blacks’ attitudes or stereotypes regarding Whites [10–12], none of this prior work has examined essentialist beliefs. Here, we present data that represent a much-needed effort toward exploring minority group members’ essentialist thinking about Whites. Key questions include: (1) What is the structure of minority group members’ essentialist thinking about Whites?; and (2) Does such essentialist thinking predict affective prejudice or preferences regarding contact with Whites?

Before presenting the data, we will review what has been learned to date about the structure of majority group members’ essentialist thinking and its attitudinal correlates. This review will provide justification for the measurement approach in our studies.

### The Structure of Essentialist Thinking

#### Natural kind and entitativity beliefs

Rothbart and Taylor [3] were pioneers in theorizing about essentialism of social categories. They explored the possibility that human beings sometimes conceptualize social categories as *natural kinds*. Natural kind categories are believed to possess an underlying essence that is fundamental to category membership, and which gives rise to category-based features. For example, the natural kind *gold* has a distinct underlying molecular structure that causes its characteristic appearance and other physical properties. Rothbart and Taylor theorized that certain social categories—especially gender and ethnic categories—are often misconstrued as natural kind categories and that this misconstrual explains why these categories play such a powerful role in generating assumptions about individual category members. Note that, in making this suggestion, Rothbart and Taylor are not concerned with the particular trait content of beliefs about groups. Rather, they are concerned with people’s naïve “theories” regarding the origin of those traits: Do people assume that group traits (or other group features) are a manifestation of a deep, underlying “nature” shared by group members (e.g., “Of course he is competitive…men are just *made* that way!”)?

Yzerbyt, Rocher, and Schadron [4] continued this theoretical thread and developed an important conceptual distinction between *natural kind beliefs* and perceived *entitativity*. Natural kind and entitativity beliefs have become the two fundamental components in many theoretical treatments of essentialism. Whereas natural kind beliefs focus on the notion of a deep, underlying basis of a category, entitativity focuses on “surface features.” In particular, entitativity refers to the extent to which a group is seen as homogenous on traits or goals (*they are all sociable*; *they all want to seize power*). In developing these ideas, Yzerbyt et al. drew from the cognitive psychology literature on the representation of categories [13], [14]. They suggested that, just like non-social categories, social categories are can be viewed in terms of prototypical features (*stereotypes/entitativity*) and in terms of explanatory theories that illuminate why those features are present (*essentialist theory/natural kind beliefs*).

A major contribution of Yzerbyt and his colleagues was the suggestion that natural kind beliefs and entitativity perceptions can be viewed as two interactive components of group representations. Indeed, conceptually separating these constructs has enabled clearer understanding of how they interact [15] and sometimes diverge [16]. Yzerbyt et al. [4] discussed at length the seminal study of Hoffman and Hurst [17] to illustrate this interaction. Hoffman and Hurst presented participants with information about the social roles and individual traits of 15 individuals from two fictional groups, the “Orinthians” and the “Ackmians.” One group was a “male-analogue”: 80% city workers and 20% child raisers. The other was a “female-analogue”: 80% child raisers and 20% city workers. Despite this difference in social roles, information provided about group members’ traits was exactly the same across the groups. Despite identical trait information, participants judged the female-analogue category as more communal and the male-analogue category as more agentic. Importantly for present purposes, this tendency to infer group traits was especially pronounced when participants were primed to think in essentialist terms, namely when the groups were described as two different species. In sum, inferences about the sharedness of group traits (entitativity) were more extreme in the presence of a natural kind conception of the groups. Martin and Parker [18] present similar results.

Haslam et al. [1] approached the topic of essentialism from a different point of departure but ended with similar conclusions to those of Yzerbyt and his colleagues. Haslam et al. aimed to illuminate the concept of essentialism by identifying the constituent features of essentialist beliefs and then empirically examining their interrelations. They reviewed core ideas from philosophy of language, philosophy of biology, critical social theory, psychological anthropology, social psychology, and cognitive psychology to determine how essentialism was conceptualized in those literatures. They found that a variety of specific features were associated with essentialism: Essentialized categories have a *natural basis* and *underlying reality*; category membership is *discrete*, *immutable*, *stable over time*, based on *necessary features*, and *highly informative about the features of category members*; and category members are *highly uniform in their features*. They had participants rate 40 social categories (e.g., vegetarians, Asians, old people, Catholics, lower-class people, etc.) on each of the nine features of essentialism they identified from the literature. They factor analyzed the resulting ratings to determine how many unique factors underlie the nine specific features of essentialism. They found a two-factor solution in which essentialist belief comprises belief in *naturalness* (categories are discrete, natural, immutable, stable, and have necessary features) and *entitativity* (members are uniform in their features, category membership is highly informative, and there is an underlying reality to the category). Haslam et al. [2] replicated this factor structure in a study focused on essentialist beliefs about women, Blacks, and gay men.

In sum, there is theoretical precedent and empirical backing for thinking of essentialism in terms of whether a group is seen as having a natural basis and whether group members are seen as highly uniform in their traits and goals.

#### A second look at entitativity: Is it really essentialism?

Going against the grain of the work just discussed, Hamilton [19] builds a compelling case that essentialism should be defined strictly and solely in terms of natural kind beliefs—belief in a deep, underlying, natural basis to a category—and that entitativity should be treated as a separate construct. Of course, Hamilton recognizes that natural kind thinking can give rise to increased perceptions of entitativity, and vice versa. On the other hand, it is also clear that strong perceptions of entitativity can stem from non-essentialistic bases, such as the perception that group members share a similar social background [20] or have made similar life choices [16]. Because of this, Hamilton persuasively argues that equating entitativity with essentialism—or including it as a defining feature of essentialism—is a conceptual error that can only lead to confusion.

Here, we follow Hamilton by defining essentialism exclusively in terms of natural kind beliefs. Recent scholarship has suggested that there might be different kinds of natural kind beliefs in relation to social groups.

#### A distinction within natural kind beliefs: Bio-somatic versus bio-behavioral essentialism

Andreychik and Gill [5] were concerned with the link between essentialism and *affective prejudice*—dislike, disrespect, animosity—toward outgroups. Surprisingly, given the intuitive expectation of a strong link, they found that the research literature provided rather mixed evidence for such a relation. For example, some scholars have found weak or flexible relations between essentialism and affective prejudice [2], [8], [21]. On the other hand, others have found positive links between essentialism and affective prejudice [6], [7]. For Andreychik and Gill, the key question was: How can we bring order to these findings and clarify when essentialism will contribute to prejudice?

Starting from yet another point of departure, Andreychik and Gill [5] examined the literature on how people reason about biological inheritance. Their rationale for examining this literature was that the notion of “essence” is typically intertwined with a notion of “underlying biology” [22], [9], and one’s underlying biology is inherited from one’s ancestors. Thus, looking at how people think about biological inheritance might illuminate how they think about the concept of “group essence.”

One paradigm for studying lay thinking about biological inheritance is the *adoption paradigm*. In an influential study, Solomon, Johnson, Zaitchik, and Carey [23] presented children and adults with stories in which an individual was born and then immediately adopted. The biological parent was described as possessing physical features (e.g., *blue eyes*) and psychological features (e.g., *outgoing*) that differed from those of the adoptive parent (e.g., *green eyes* and *shy*). Results indicated that by age 7 (and continuing into adulthood) participants predicted that the child would resemble the birth parent on somatic features, but would resemble the adoptive parent on psychological features. For Andreychik and Gill, the key point was that when people think about what is transmitted from ancestors they make a crisp distinction between *somatic features* and *psychological features* [24], [25]. Notably, this pattern of reasoning is not unique to formally educated North American subjects [26–29]. Indeed, when asked about the adoption scenario the Vezo people of Madagascar—a thoroughly non-Westernized culture that lacks formal education—make precisely the same judgments as do residents of the northeastern U.S. [26].

Gil-White’s [28] work with Mongol participants showed that essentialist thinking about *groups* also includes this distinction between inheritance of somatic versus psychological features. That is, nearly 100% of his participants said that a child born of Kazakh parents and adopted by Mongol parents would *be* a Kazakh and would *physically resemble* Kazakhs. Yet, a significant number of these same participants said that the child would *act* like a Mongol, showing no tendency to inherit the mental or behavioral traits of Kazakhs (despite *looking like* and *being*, in the eyes of participants, a Kazakh). Andreychik and Gill [5] suggested that people who reason in this way are showing *bio-somatic essentialism*: Belief that there is a group essence passed from parent to child that creates a physical reality to social groups, a reality evident in the ostensibly shared appearance of group members.

Whereas many of Gil-White’s [28] participants held *only* bio-somatic essentialist beliefs (showing the pattern of judgment noted above), other participants provided evidence of what Andreychik and Gill [5] frame as a different, more encompassing type of essentialist thinking. That is, these participants not only believed that the adopted child would *physically be* a Kazakh, but that he would also *think and behave like a Kazakh*. They believed that a Kazakh group essence would be present in him that would give rise to Kazakh-typical cognitive and behavior patterns, despite his having no contact with the Kazakh group. Andreychik and Gill called this thinking *bio-behavioral essentialism*: Belief that there is a group essence passed from parent to child that creates a mental/behavioral reality to social groups, a reality evident in the characteristic cognitive, emotional, and behavioral style of group members.

Andreychik and Gill [5] note that both bio-somatic and bio-behavioral essentialist beliefs are natural kind beliefs. They differ only in terms of which group features—somatic, psychological—are seen as being generated by the group essence. Consistent with their theorizing, Andreychik and Gill showed that bio-somatic and bio-behavioral essentialism emerge as two independent factors in a factor analysis of items that reflect both types of thinking.

#### Summary

Some researchers have suggested that essentialism consists of both natural kind and entitativity beliefs. Others have rejected this idea and suggested that essentialism consists of natural kind beliefs, and entitativity is a separate (albeit very important) concept. Finally, recent work has suggested the existence of two different types of natural kind beliefs: Bio-somatic and bio-behavioral essentialism.

### Attitudinal Correlates of Essentialist Belief

Beyond examining belief structure, researchers have explored whether essentialist belief is predictive of affective prejudice and attitudes regarding intergroup contact. Andreychik and Gill [5] showed that bio-behavioral essentialism is strongly predictive of affective prejudice, whereas bio-somatic essentialism is unrelated to affective prejudice. Thus, thinking of a group as a biologically real entity whose reality is “merely physical” is insufficient to energize prejudice; rather, the ostensible biological reality of the group must extend to encompass group character in order to evoke prejudice. Andreychik and Gill further argued that their distinction between two types of essentialism—i.e., bio-somatic, bio-behavioral—lends order to prior work on essentialism and prejudice. For example, they noted that Jayaratne et al. [6] and Keller [7]—both of whom found a connection between essentialism and affective prejudice—were clearly assessing bio-behavioral essentialism. On the other hand, Haslam et al. [2]—who found no relation between naturalness beliefs and prejudice—used a measure that appears to conflate bio-somatic and bio-behavioral essentialism.

Rather than focusing on affective prejudice, Williams and Eberhardt [9] looked at Whites’ openness to intergroup contact with Blacks. They showed that biological conceptions of racial categories resulted in diminished desire for intergroup contact. Although their goal was to measure and manipulate bio-somatic essentialism, it is not entirely clear whether they were successful. The reason is that they did not explicitly control for the possibility that their measures and manipulations also targeted bio-behavioral essentialism to some extent. In any event, their work did demonstrate a link between essentialism and reduced desire to connect with the outgroup.

## The Present Study

Conceptual and statistical analysis have suggested that essentialism can be understood in terms of two different kinds of natural kind belief: Bio-somatic and bio-behavioral. Will a similar belief structure be evident in minority group members’ essentialist thinking about Whites? This is the first question that will be addressed by the data presented below. Based on Hamilton’s [19] arguments, we will leave entitativity out of our analysis, as it is not necessarily a component of essentialist thinking.

Research has also suggested that bio-behavioral essentialism is uniquely predictive of affective prejudice toward social groups, and that biological conceptions of racial groups predict diminished desire to interact across group lines. Will similar relations between essentialism and attitudes be found in minority group members’ responses to Whites? This is the second question that will be addressed by the data presented below.

Although a primary goal when embarking on this line of research was to gather data regarding an issue that is strikingly understudied (i.e., essentialist thinking within non-dominant minority groups), we did enter with some predictions in mind. First, we expected to replicate within a minority group the structure of essentialist thinking uncovered by Andreychik and Gill [5]. This expectation was grounded in prior research which suggests that the distinction between inheritance of somatic versus psychological features reflects something very basic about human cognition, appearing in the thinking of peoples as diverse as middle-class Westerners, semi-nomadic Mongol shepherds, and the Vezo people of Madagascar.

We note, however, that we were also able to generate an alternative prediction: A unidimensional structure of essentialism—no distinction between bio-somatic and bio-behavioral—within the minority group. Derivation of this prediction begins with an assumption that essentialist belief starts out with a single-factor structure: There is an underlying biological reality to groups which generates *all* group-linked features (i.e., both appearance and behavior). Indeed, historical analysis suggests that essentialist thinking did, in fact, start out with such a unidimensional structure [30]. Given this starting point, it is possible that Whites’ tendency to distinguish between bio-somatic and bio-behavioral essentialism arose as a “pruning” of the original, singular essentialism construct. The pruning might have been instigated by historically recent social pressures to avoid derogatory thinking about minority groups that could be used to rationalize their oppression. Of course, bio-behavioral essentialist beliefs—with their implication of biologically determined (inferior) character—seem most clearly relevant for justifying oppression. Thus, such beliefs would be most widely rejected under this scenario. On the other hand, bio-somatic essentialist beliefs could be maintained because they are not inherently oppression-supporting. This argument is consistent with Andreychik and Gill’s [5] pattern of data in which bio-somatic essentialism is endorsed far more strongly by Whites than is bio-behavioral essentialism, and bio-somatic essentialism is unrelated to both bio-behavioral essentialism and to prejudice. Because minority group members have presumably received less social pressure to reject beliefs that justify oppression (because they are not in a position to oppress), their essentialist thinking might be “unpruned” and thus more likely to reflect the “original” unidimensional structure of essentialist thought.

We also expected to replicate Andreychik and Gill’s finding that bio-behavioral essentialism is uniquely important for predicting negative attitudes, whereas bio-somatic essentialism is unrelated to negative attitudes. A relatively elaborate statement of the theoretical basis for this prediction is offered by Andreychik and Gill. Suffice it to say here that bio-behavioral essentialism links group members decisively and inescapably to whatever negative traits one associates with the group (e.g., “White people think too highly of themselves and are unfair to other groups"; [12]) thereby intensifying negative affective reactions. We can call this the *cementing theory* of essentialism and prejudice. In contrast to bio-behavioral essentialism and its capacity to cement a group to unlikable traits, bio-somatic essentialism links group members only to having particular types of bodies and thus leaves open the possibility that any unlikable behavioral traits are malleable.

As with the structure of essentialism, we also found it possible to generate an alternative prediction here: Essentialist beliefs of the minority group will be unrelated to prejudice. Derivation of this prediction comes from the theoretical perspective of Morton, Postmes, Haslam, and Hornsey [21]. Those authors provide evidence supporting their prediction that essentialism will only generate prejudice when the essentialist beliefs belong to a dominant group that feels its social position is threatened by a subordinate group. When the ingroup’s dominant position is threatened, essentialist beliefs about the outgroup will contribute to prejudice; otherwise, essentialist beliefs will be unrelated to prejudice. We can call this the *status maintenance theory* of essentialism and prejudice. Because minority groups in the U.S. are non-dominant and because they presumably do not have fears that Whites are trying to take over their position in society, the perspective of Morton et al. suggests that essentialist beliefs of the minority group will be unrelated to prejudice.

Our study was approved by Lehigh University’s Institutional Review Board (ID# 421311–3). For each study, participants provided consent by clicking an “agree” button on a computerized consent form. If they clicked “disagree,” they did not receive any of our measures. The consent form was approved by Lehigh University’s IRB.

### Method

#### Participants

Participants were 270 minority group individuals who were recruited using the “panel” feature of Turk Prime [31], which enabled us to recruit only African American participants. Data from one participant was deleted because he provided the same rating for every question, and data from twenty-one additional participants were deleted because they were bi-racial/bi-cultural. This left a final sample of 248 (150 female) all of whom self-identified exclusively as African American. Participants ranged in age from 19 to 66 (*M* = 34, *SD* = 10.6). Eleven percent of participants had completed only high school, 30% had completed less than two years of college, 12% a two-year college degree, 36% a four-year college degree, and 11% a graduate or professional degree. Participants were originally from two studies (*N* = 120, *N* = 150) conducted four days apart, but the samples were combined into one after finding identical results across the two studies.

#### Measures

All measures are available in S1 Measures. Participants completed the bio-somatic essentialism and bio-behavioral essentialism measures of Andreychik and Gill [5]. The measures were reworded to make “White people” rather than “African Americans” the focal group. Participants completed a measure of affective prejudice toward Whites, based on the measure used by Andreychik and Gill to assess affective prejudice. In addition, they completed a measure of preferences regarding contact with Whites, modeled after the Other-Group Orientation subscale of the Multigroup Measure of Ethnic Identity [32], [9]. All measures were completed using a five-point response scale with endpoints labeled *Strongly Disagree* (1) and *Strongly Agree* (5).

#### Procedure

Participants accessed and completed all measures as part of a single “Human Intelligence Task” on Amazon’s Mechanical Turk. The order of measures was the same as their order of presentation in the Measures section above.

### Results and Discussion

#### Structure of essentialist thinking

Following the analytic approach of Andreychik and Gill [5], we submitted the four bio-somatic and four bio-behavioral essentialism items to a principal components analysis with varimax rotation. Replicating the results of Andreychik and Gill, this analysis revealed two components with eigenvalues greater than 1 (i.e., 3.12 and 1.74, accounting for 39.02% and 21.79%, respectively, of the item variance). Loadings of each item on each component can be seen in Table 1. As can be seen there, the present study perfectly replicates the two factor structure that Andreychik and Gill found for Whites’ essentialist thinking about Blacks. In addition to replicating that factor structure, the pattern of means for bio-somatic essentialism (*M* = 3.6, *SD* = .72, α = .76) and bio-behavioral essentialism (*M* = 2.3, *SD* = .95, α = .84) also paralleled what Andreychik and Gill found for Whites’ essentialist thinking about Blacks. That is, bio-somatic essentialism was endorsed significantly more than was bio-behavioral essentialism, paired-samples *t*(247) = 20.16, *p* < .001 (Cohens *d*_rm_ = 1.39).

**Table 1:** Factor loadings of bio-somatic and bio-behavioral items.

	Component 1	Component 2
**Bio-Somatic items**		
Biological basis of group appearance	.07	.79
Physical differences have biological basis	-.01	.82
Hard for Whites to be “African American” in appearance	.21	.68
Somatic differences are stable across time	.14	.60
**Bio-Behavioral items**		
Biological basis of group behavior and thinking	.83	.18
Differences in life trajectories have biological basis	.83	.11
Hard for Whites to be “African American” in thought/beh	.83	.10
Behavioral differences are stable across time	.76	.06

#### Essentialist beliefs and intergroup attitudes

Are minority group members’ essentialist beliefs meaningful—in terms of enabling prediction of attitudes—in the same way as Whites’ essentialist beliefs? To test this, first we averaged together the three *prejudice* items (*M* = 1.75, *SD* = .83, α = .87). Next, because they appear heterogeneous, we submitted the intergroup contact items to a principal components analysis with varimax rotation. This revealed two principal components, one of which included items 1–4 [actively seek contact, apathetic about contact (reverse scored)] and the other of which included items 5–6 [actively reject contact]. Thus, after reverse scoring items 3–4, we computed separate indicators of *contact rejection* (*M* = 1.70, *SD* = .83, α = .87) and *contact seeking* (*M* = 3.48, *SD* = .85, α = .88). The tendency for positive and negative assessments of a stimulus to be somewhat independent is well-established in the attitudes literature [33], and arguably reflects the fact that attitudes are often ambivalent (e.g., “In some ways, I am attracted to interacting with the outgroup; In other ways, I am repulsed.”).

Next, we utilized multiple regression analysis to determine the unique contribution of each essentialism dimension to our attitude indicators. Results can be seen in Table 2. As can be seen there, results for affective prejudice replicated what Andreychik and Gill [5] found for Whites’ essentialist thinking about Blacks: Bio-behavioral essentialism predicted increased affective prejudice, whereas bio-somatic essentialism did not. The results for rejecting contact with Whites paralleled those for affective prejudice. Finally, the results for seeking contact were somewhat different: Neither dimension of essentialism was associated with positive desire for contact with Whites. Thus, bio-behavioral essentialism predicted the most expressly negative of our attitudinal indicators (i.e., affective prejudice, active rejection of contact), and neither essentialism dimension predicted the positive attitude indicator (i.e., active seeking of contact).

**Table 2:** Unique contribution of essentialism components to attitudes: Multiple regression analyses.

	Predictor Variables	
	Bio-Somatic Essentialism	Bio-Behavioral Essentialism
Outcome variables:		
Affective Prejudice	β = .02	β = .20**
	[*t* = .32, Cohen’s *f*^2^ = .00]	[*t* = 3.15, Cohen’s *f*^2^ = .04]
Reject Contact	β = .02	β = .24***
	[*t* = .29, Cohen’s *f*^2^ = .00]	[*t* = 3.72, Cohen’s *f*^2^ = .06]
Seek Contact	β = -.04	β = -.08
	[*t* = -.58, Cohen’s *f*^2^ = .00]	[*t* = -1.16, Cohen’s *f*^2^ = .00]

*Note*. Degrees of freedom for all *t*-tests is 245.

**p < .01,

***p < .001

## General Discussion

Essentialism in perceptions of social groups has garnered a significant amount of research attention. This is because essentialist beliefs presumably give rise to negative intergroup attitudes and interactions. When researchers have studied essentialist beliefs about real social groups, they have overwhelmingly assessed how dominant Caucasian majorities think about non-dominant minority groups. To correct this imbalance, we proposed to address two questions in the present article: (1) What is the structure of minority group members’ essentialist thinking about Whites?, and (2) Does such essentialist thinking predict affective prejudice or preferences regarding contact with Whites? We now have our answers.

The study presented here provides evidence that Blacks’ essentialist conceptions of Whites possess a conceptual structure and a mean structure that is virtually identical to that found for Whites’ essentialist conceptions of Blacks [5]. That is, Blacks’ essentialist conceptions are characterized by a distinction between bio-somatic and bio-behavioral essentialism, and Blacks endorse bio-somatic essentialism to a significantly greater degree than bio-behavioral essentialism. These results provide further support for Andreychik and Gill’s [5] argument that insight into essentialism can be gained from forging connections between the study of essentialism and the literature on how people think about biological inheritance [23–29]. Indeed, the present studies add to a diverse body of evidence suggesting that the distinction between inheritance of somatic versus psychological features runs deep within the human social-cognitive conceptual repertoire: The distinction is apparent when White Americans think about Black Americans, when Black Americans think about White Americans, when Mongol shepherds think about Kazakhs, and when the Vezo people of Madagascar think about themselves and their neighbors from different ethnic and cultural groups. The present data argue against the possibility, raised above, that dominant group members have “pruned” their essentialist beliefs in a way that non-dominant group members are unlikely to have done. Rather, the conceptual structure of their essentialist thinking is quite similar.

As noted above, work on essentialism has not only examined its conceptual structure, but has also sought to identify which particular essentialism concepts contribute to negative intergroup attitudes. The present studies provide evidence regarding this issue and the evidence is, again, highly consistent with the findings of Andreychik and Gill [5]. Specifically, bio-behavioral essentialist beliefs are predictive of indicators of blatant bias such as affective prejudice and active rejection of contact with the outgroup, whereas bio-somatic essentialist beliefs are unrelated to bias. The fact that we find a relation between bio-behavioral essentialism and prejudice within a non-dominant minority group is consistent with the *cementing theory* (see above) and difficult to reconcile with the *status maintenance theory* of essentialism (see above). This does not mean that status maintenance concerns are irrelevant to the essentialism/prejudice link, but rather that such concerns are not *necessary* to bring an essentialism/prejudice link into existence.

Although bio-somatic essentialism is unrelated to indicators of animus, Andreychik and Gill provided evidence that bio-somatic essentialism is predictive of some types of beliefs, such as belief in the importance of using race information in the context of medical practice (e.g., recognizing that “Black bodies” might have certain disease susceptibilities that “White bodies” do not). Future research is needed to explore additional consequences of bio-somatic essentialism. One plausible prediction is that those with strong bio-somatic essentialist beliefs will be more likely to show cross-race recognition deficits [34, 35], with an increased tendency to see “them” as “all looking alike.” This would be the case because their “theory” of an essential basis for group appearance hinders perception of within-group physical diversity.

The present work, paired with the work of Andreychik and Gill [5], has implications for changing intergroup attitudes. It is notoriously difficult to persuade people to stop thinking of human diversity in terms of *racial (natural) kinds*, even when those people are social scientists with high levels of exposure to the relevant evidence [30]. The “biological reality of race” is a powerful illusion. Of course, social scientists should chip away at the illusion, while recognizing that this might be a very long-term project. Intriguingly, our work suggests that total destruction of that illusion is not necessary to accomplish the arguably more immediate goal of prejudice reduction. The reason is that racial kind concepts *per se* do not contribute to prejudice as long as those concepts refer merely to group morphology and not to group character. Thus, future research on prejudice reduction might profitably explore how people’s racial kind concepts could be reined in to reflect mere bio-somatic essentialism, excising any prejudice-evoking bio-behavioral essentialist components. Practically speaking, this might be easier to accomplish than convincing people that there is no biological reality to race whatsoever. We suggest that one effective tool for excising bio-behavioral essentialism might be to provide people with *alternative explanations for group character*. For example, if people came to recognize how group traits and group worldviews are shaped by the shared life histories of group members [36], then essentialist explanations for those traits and worldviews become unnecessary. We would predict, of course, that such historical explanations would leave bio-somatic essentialism perfectly intact. Nevertheless, the undermining of erroneous bio-somatic essentialist thinking could remain an important long-term goal (see [37] regarding the errors of racial kind thinking), but reducing animosity—via specifically targeting bio-behavioral essentialist beliefs—seems to us a more pressing concern.

The present studies contribute to a small body of work that has examined essentialist thinking among minority group members. Prior work differs from the present study in the sense that none of it has examined minority group members’ essentialist beliefs *about the majority group* and linked those beliefs to *negative attitudes toward that majority group*. Also, prior work has not considered the distinction between bio-somatic and bio-behavioral essentialism, and how this distinction is important for predicting negative attitudes.

For example, Verkuyten and Brug [38] looked at essentialist beliefs and support for multiculturalism within minority groups. For minority group members, support for multiculturalism is a “pro-ingroup” rather than “anti-outgroup” attitude. Verkuyten and Brug measured essentialist thinking about the ingroup and outgroup among Turkish, Moroccan, and Surinamese minorities in the Netherlands. They found that only *ingroup* essentialism of the minority group (i.e., their conceptions of their own group) predicted increased support for multiculturalism, whereas *outgroup* essentialism (i.e., minority group conceptions of the ethnic Dutch majority) was unrelated to such support. Interestingly, among the ethnic Dutch majority group, only *outgroup* essentialism (i.e., conceptions of the minority groups) predicted reduced support for multiculturalism, whereas ingroup essentialism was unrelated to such support. Thus, this work suggests that the way people think about the minority group is particularly important, whereas the way they think about the dominant group is not. This contrasts with our finding that essentializing the dominant group is associated with negative attitudes toward that dominant group. The work of Verkuyten and Brug also suggests that minority *ingroup* essentialism can function as a means of supporting or even celebrating that ingroup (e.g., via increasing support for multiculturalism policies).

This notion that essentialist beliefs about the ingroup can serve positive functions for a minority group was also important for Morton and Postmes [39]. They examined ingroup essentialism among sexual minorities (i.e., gay, lesbian, bisexual, queer) and tested the hypothesis that such essentialism would be increased under conditions of feeling like an “invisible minority” (due to an urge to assert, “Hey, we are real and must be recognized!”) but not under conditions of feeling like a “persecuted minority” (due to an urge to de-emphasize an identity that makes one a “target”). Morton and Postmes manipulated whether members of the sexual minority groups recalled instances of being invisible or instances of being persecuted and found that, as predicted, ingroup essentialism—in particular the notion of a biological basis of sexual orientation—increased under conditions of invisibility salience but not under conditions of persecution salience. They also found that this increased essentialism was associated with positive expectations about social changes that will be good for sexual minorities. Thus, similar to the work of Verkuyten and Brug [38], minority ingroup essentialism seems linked to a positive assertion of identity in this study.

Finally, other researchers have examined how essentialist belief within a minority group can hinder their identification with the majority culture within which they live. For example, No, Hong, Liao, Lee, Wood, and Chao [40] focused on the impact of holding a broad essentialist racial theory (i.e., “Race determines traits and abilities”) among Asian Americans. They presented diverse sources of evidence which converged to suggest that essentialist theories reduce the tendency for minority group members to identify with elements of majority group culture. That is, essentialist beliefs among Asian Americans predicted greater perceptions of personality differences between Asian and White Americans, reduced tendency to expressly identify with (White) American culture, and reduced tendency to be affected by priming with stimuli associated with (White) American culture. Bastian and Haslam [41] presented similar findings regarding essentialism and hindered acculturation within a minority group, although in their study minority group members’ resistance to identifying with majority group culture was better predicted by entity theories [42] than by a broader measure of essentialist beliefs.

## Conclusion

Minority group members’ essentialist beliefs about Whites reflect the same two-component structure (bio-somatic, bio-behavioral) uncovered for Whites’ beliefs about minorities. Also, minority group members, like Whites, endorse bio-somatic essentialism significantly more than they endorse bio-behavioral essentialism. And, replicating what Andreychik and Gill [5] found for Whites’ essentialist beliefs, bio-behavioral essentialist beliefs of the minority group predict blatant indicators of bias, whereas bio-somatic essentialist beliefs are unrelated to bias. Of course, the present findings reflect only Blacks’ views of Whites. The perspectives of other minority groups should be examined. Finally, we encourage future research to explore how bio-behavioral essentialism can be undermined within all groups, and we suggest that one promising avenue might be to acquaint people with alternative, historical explanations for group character.

## Supporting Information

S1 Measures Measures used in the studies. (DOCX)
