# Supplementary material for: When the Minority Thinks “Essentially” Like the Majority: Blacks Distinguish Bio-Somatic from Bio-Behavioral Essentialism in Their Conceptions of Whites, and Only the Latter Predicts Prejudice
Source: PLoS One. 2016 Aug 4;11(8):e0160086. doi: 10.1371/journal.pone.0160086 (PMC4973969; doi:10.1371/journal.pone.0160086)
Supplement: S1 Measures — (DOCX) [file pone.0160086.s001.docx]

**All measures were completed using a five-point response scale with endpoints labeled

*Strongly Disagree* (1) and *Strongly Agree* (5).

**Bio-Somatic Essentialism (Andreychik & Gill, 2015)**

(1) There are underlying genetic/biological factors that make White people similar to

one another—and different from African Americans—in terms of their *physical characteristics*.

(2) Differences in hair color and texture, eye color, and facial features between Whites

and African Americans can be understood in terms of underlying biological factors.

(3) Because of the genetic/biological factors that determine their physical characteristics, it

is quite difficult for most Whites to be highly “African American in appearance” or for

most African Americans to be highly “White in appearance.”

(4) Because of their underlying biological/genetic basis, differences in physical

characteristics between Whites and African Americans have probably been roughly the

same for a long time and will continue into the foreseeable future.

**Bio-Behavioral Essentialism (Andreychik & Gill, 2015)**

(1) There are underlying genetic/biological factors that make Whites similar to

one another—and different from African Americans—in terms of their *behavior and*

*thinking* (e.g., their personality traits, attitudes, abilities).

(2) Differences in the life trajectories (e.g., education, careers) of Whites vs. African Americans can

be understood in terms of the different underlying biological capacities of each race.

(3) Because of the genetic/biological factors that determine their behavior and thinking, it is quite

difficult for most Whites to “think and act like African Americans” or for African Americans to

“think and act like Whites.”

(4) Because of their underlying biological/genetic basis, differences in behavior and thinking (e.g.,

personality traits, attitudes, abilities) between Whites and African Americans have probably

been roughly the same for a long time and will continue into the foreseeable future.

**Affective Prejudice**

(1) I dislike White people.

(2) White people disgust me.

(3) I often find myself feeling angry at White people.

**Attitude Towards Cross-Race Contact**

(1) I like interacting with White people.

(2) I seek relationships with White people.

(3) I do *not* feel much motivation to seek relationships with White people.

(4) I do *not* put much effort into seeking relationships with White people.

(5) I try hard to avoid contact with White people.

(6) Keeping my distance from White people is important to me.
